# Addiction medicine in modern contexts: hot topics from around the globe

**DOI:** 10.1192/bji.2022.13

**Published:** 2022-08

**Authors:** Hussien Elkholy

**Affiliations:** Associate Professor of Psychiatry, Neurology and Psychiatry Department, Faculty of Medicine, Ain Shams University, Cairo, Egypt. Email: h.elkholy@med.asu.edu.eg

**Keywords:** Addiction, tramadol, captagon, khat, gaming disorder

## Abstract

Addiction is a chronic yet treatable disorder. Patterns of addiction, whether substance related or behavioural, vary among countries and regions. Addiction medicine practice and approaches used in management are not only different from one country to another but are influenced by other factors, including environmental ones. The COVID-19 pandemic is one of the major environmental changes that had an impact on addiction. In this editorial, light will be shed on three articles covering recent updates in addiction medicine, ranging from types of substances and service provision to inclusion of gaming disorder in ICD-11.

The American Society of Addiction medicine defines addiction as a treatable, chronic medical disease involving complex interactions between brain circuits, genetics, the environment and an individual's life experiences. People with addictions use substances or engage in behaviours that become compulsive and often continue despite harmful consequences.^1^ Addiction as a term is usually associated with substance use and dependence, which in turn is associated with a significant amount of stigma. However, addiction extends beyond alcohol and other substances, to include certain behaviours when they become harmful, for example gaming disorder. As the definition implies, addiction is a chronic disease yet treatable. Yet, the above-mentioned stigma and other factors play important roles in the understanding of the disease, treatment approaches and their availability. It is also noteworthy that the profile of drugs of misuse differs from one country/region to another. Moreover, other environmental factors, such as the COVID-19 pandemic, may have an impact on services and even the pattern of drug use.

The current issue of *BJPsych International* includes three articles on the theme of addiction, which I introduce below.

## COVID-19 pandemic: use of harm reduction and abstinence-based approaches in different countries

Harm reduction is an umbrella term used for a set of ideas, interventions and practical strategies aimed at reducing negative consequences associated with substance use and other health behaviours, whereas abstinence refers to complete cessation of substance use. In general, abstinence-based models have dominated treatment programmes globally and have been an inherent component of different programmes. The COVID-19 pandemic has significantly affected treatment services for people with substance use disorders (SUDs). Based on the perspectives of service providers from eight countries, the first article on our theme, by Narasimha et al,^2^ discusses the impact of the pandemic on SUD treatment services. Although many countries quickly adapted in provision of harm reduction services by changes in policy and service delivery, some adopted a forced abstinence-based strategy. Similarly, disruption of abstinence-based approaches has been reported.

## Substance use in the Eastern Mediterranean region

The Eastern Mediterranean Region, given its special geopolitical situation and internal/external conflicts, faces an increase in illegal activities such as drug production and trafficking, highlighting the need for a comprehensive understanding of the substance use situation. The prevailing situation of war, insurgencies, political conflict and civil unrest in many countries of the region has dramatically influenced all aspects of substance use, from production and trafficking to availability and pattern of use. This is compounded by the long-standing position of this region as one of the largest opium production sites globally. In the second article on our theme, Mohaddes Ardabili et al^3^ shed light on patterns of substance use in the region.

## Inclusion of gaming disorder in ICD-11: global needs

As mentioned above, it is not only substances that can become addictive but behaviours as well. The World Health Organization has added gaming disorder to ICD-11 as a clinical condition associated with distress or interference with personal functioning. This inclusion leads to clinical and public health benefits, such as harmonising terminology, offering clinical landmarks and improving monitoring capabilities and data comparability. Training health professionals to identify and manage gaming disorder is a key challenge for countries. In the final article on our theme, Long et al^4^ compile opinions from different countries around the world on their state of preparedness and needs to tackle this issue. They conclude that collective international efforts are imperative to develop high-quality training tools that can be adapted for use by healthcare providers in various cultural settings. Moreover, the cost-effectiveness of scaling up interventions deemed to be a crucial consideration in the management of other mental health conditions should also apply to gaming disorder.

## Data availability

Data availability is not applicable to this article as no new data were created or analysed in this study.
